# Exploring machine learning for audio-based respiratory condition screening: A concise review of databases, methods, and open issues

**DOI:** 10.1177/15353702221115428

**Published:** 2022-08-16

**Authors:** Tong Xia, Jing Han, Cecilia Mascolo

**Affiliations:** Department of Computer Science and Technology, University of Cambridge, 15 JJ Thomson Avenue, Cambridge CB3 0FD, UK

**Keywords:** Respiratory abnormity, respiratory sound, artificial intelligence, machine learning, auscultation, automatic disease diagnosis

## Abstract

Auscultation plays an important role in the clinic, and the research community has been exploring machine learning (ML) to enable remote and automatic auscultation for respiratory condition screening via sounds. To give the big picture of what is going on in this field, in this narrative review, we describe publicly available audio databases that can be used for experiments, illustrate the developed ML methods proposed to date, and flag some under-considered issues which still need attention. Compared to existing surveys on the topic, we cover the latest literature, especially those audio-based COVID-19 detection studies which have gained extensive attention in the last two years. This work can help to facilitate the application of artificial intelligence in the respiratory auscultation field.

## Impact Statement

With the rapid progress of artificial intelligence for auscultation, comes the pressing need to compile a compendium of existing works and present recent advances. This concise review aims to guide researchers who are new to either artificial intelligence or respiratory pathology, and shed light on the application of machine learning in remote respiratory condition screening. This review also seeks to inspire more work emerging from the intersection of artificial intelligence and respiratory health.

## Introduction

The respiratory system is one of the major components of the human body, with the primary and very important function of gas exchange to supply oxygen to the blood.^1^ It consists of two respiratory tracts: (1) the upper tract including the nose, nasal cavities, sinuses, pharynx and the part of the larynx above the vocal folds and (2) the lower tract including the lower part of the larynx, the trachea, bronchi, bronchioles and the lung.^2^ The upper track also works for pronunciation: generating sounds and speech. Inflammation, bacterial infection, or viral infection of the respiratory tracts can lead to respiratory diseases.^3,4^ Illnesses caused by inflammation include chronic conditions such as asthma, cystic fibrosis, and chronic obstructive pulmonary disease (COPD). Acute conditions, caused by either bacterial or viral infection, can affect either the upper or lower respiratory tract like pneumonia, influenza, and the COVID-19. As reported, the respiratory disease affects one in five people, and it is the third biggest cause of death in England.^5^ Early detection of respiratory tract infections can lead to timely diagnosis and treatment, which can result in better outcomes and reduce the likelihood of severe complications.

Notable penetration of smart devices brings new opportunities to enable individual health sensing regardless of the existing location, time, and other constrains.^6,7^ The advance of artificial intelligence (AI) further enhances the promise of automatic disease detection from the collected bio-signals.^8,9^ Particularly, because of the nature and location of the underlying inflammation due to various diseases in the respiratory system, audible changes can be identified as diagnostic signals. Herein, AI-powered auscultation via respiratory sounds collected by electronic stethoscopes and smartphones has received massive attention for its high flexibility and scalability.^10^ Traditional auscultation is usually done by respiratory physicians while training those experts to be qualified is costly in both time and money. Moreover, to be diagnosed, individuals need to go to the hospital or clinical venues, which increases clinical expenses and the risk of virus exposure. On the contrary, automatic auscultation can reduce the burden on medical resources and expedite respiratory condition screening outside hospitals. Examples include the recently developed COVID-19 screening applications where acoustic models are studied for remote COVID-19 testing.^11^ Another representative example is *ResApp*, an app founded in 2014 in Australia, which is able to detect sleep apnoea using overnight breathing and snoring sounds recorded on a smartphone placed on the bedside table.^12^

Behind those applications, audio signal processing techniques and machine learning (ML) algorithms hold the key to an accurate diagnosis. The widely adapted ML approaches mainly encompass two types: hand-crafted feature-based ML and end-to-end deep learning. For feature-based ML models, temporal especially prosodic features including pitch, duration, intensity, the harmonics-to-noise ratio (HNR), jitter, and shimmer are widely used to detect unhealthy sounds.^13^ In addition, spectral features from the log Mel spectrogram are devised and show promising performance in a series of relevant applications.^14–17^ Those features are used as the inputs of subsequent classifiers for diagnosis. For end-to-end deep learning methods, audio waves or corresponding spectrogram are directly fed into deep neural networks which output the predictions.^18,19^

Feature-based ML models are often explainable, but the performance is hardly satisfactory due to the difficulty in identifying distinguishable hand-crafted acoustic features for a specific respiratory condition. Compared to feature-based ML models, deep learning models do not depend on explicit feature engineering, so they usually present more powerful capability of modeling audio-disease relations with the premise of massive training data. The latest state-of-the-art audio-based respiratory condition screening methods are mainly deep learning based, covering convolutional neural networks (CNNs),^32,61^ recurrent neural networks (RNNs),^59,60^ and Transformer neural networks.^41,79^ Those models have demonstrated favorable performance in detecting COPD, asthma, and other respiratory conditions.

In this article, we plan to compile a list of existing publicly available respiratory sound databases and illustrate some representative ML and deep learning methods. We hope this can provide a general view for both model developers and respiratory physicians to inspire more interdisciplinary studies. Particularly, different from previous relevant reviews, we include the latest sound-based COVID-19 detection research. Moreover, we conclude some unsolved challenges with potential solutions as future works, which are under-looked at the current stage but are of critical importance to be investigated for the reliable deployment of automatic respiratory condition screening applications.

## Data overview

ML is data-driven, with model training and evaluation depending on real-world data sets. However, clinical data collection is usually not trivial due to privacy concerns and annotation costs. To advance the model development for computer scientists and to facilitate more data collection from clinical trials, we present some main characteristics of publicly accessible respiratory sound databases, with a summary in Table 1.

**Table 1.** An overview of respiratory condition audio databases.

Data set	Year	#Sam. (#Sub.)	Sounds	Device	Respiratory conditions	Annotation
*Pertussis* ^16^	2016	38 (38)	Cough	Microphone	Pertussis, asthma, croup, BRON	Self-report
*ICBHI* ^20^	2017	6898 (126)	Lung sounds, breathing	Stethoscope, microphone	Cycle-level: crackle, wheeze; subject-level: COPD, LRTI, URTI	Expert-label
*Pfizer* ^21^	2018	6593 (unknown)	Audio	Microphone	Presence of respiratory sickness	BMAT
*Stethoscope* ^22^	2021	336 (112)	Lung sounds	Stethoscope	Cycle-level: inhalation, exhalation, crackle, wheeze; subject-level: asthma, COPD, BRON, heart failure, lung fibrosis	Expert-label
*HF Lung V1* ^23^	2021	9765 (279)	Lung sounds	Stethoscope	Cycle-level: inhalation, exhalation, wheeze, stridor, rhonchus, DAS; subject-level: acute respiratory failure, COPD, pneumonia, and so on	Expert-label
*Virufy* ^24^	2021	121 (16)	Cough	Microphone	COVID-19, asthma, diabetes, symptoms, and so on	COVID-19 PCR
*Covid19-cough* ^25^	2021	1324 (unknown)	Cough	Microphone	COVID-19	Self-report clinical verify
*COUGHVID* ^26^	2021	27,550 (unknown)	Cough	Microphone	COVID-19, with or without other respiratory conditions, with or without symptoms	Self-reported expert-label
*Tos COVID-19* ^27^	2022	143,351 (unknown)	Cough	Microphone	COVID-19 severity, symptoms	Clinical verify
*Coswara* ^28^	2022	2747 (unknown)	Breathing, cough, voice	Microphone	COVID-19, current health status, and the presence of comorbidity	Self-report
*COVID-19 Sounds* ^29^	2021	53,449 (36,116)	Breathing, cough, voice	Microphone	Sample-level: COVID-19, symptoms; subject-level: medical and smoking history	Self-report

BRON: bronchiolitis; COPD: chronic obstructive pulmonary disease; LRTI: lower respiratory tract infection; URTI: upper respiratory tract infection; PCR: polymerase chain reaction.

#Sam. (#Sub.) presents the reported data size with the number of unique subjects who contributed the data. We display the size of the data released in the labeled year; however, it needs to be noted that some data collection is still ongoing and data size might be increased later on. Lung sounds were acquired by stethoscopes from the chest wall, while other sounds were collected by varied devices with microphones. Self-reported or clinically validated respiratory conditions are concluded for various study purposes.

### Respiratory abnormality database

One of the easiest explorations of computerized respiratory sounds dates back to 2016,^16^ when researchers utilized cough sounds from YouTube to diagnose *pertussis*. This database is small, that is, 38 recordings with a duration between 10 and 169 s. Those recordings were from 38 subjects: 20 patients with pertussis cough, 11 with croup and other types of cough, and 7 with cough containing wheezing sounds corresponding to other diseases such as BRON (bronchiolitis) and asthma. Of the 38 subjects, 14 are infants aged 0–2 years, 18 are children aged 3–18 years, and 6 are adults aged over 19 years. Given the limited number of samples, despite its uniqueness, this database is not suitable for modern ML model development and validation. Yet, it is the only public database with pertussis labels. Larger pertussis-related data sets are still to be gathered and made public: this would greatly enhance the automatic detection of pertussis research.

Later on, two *challenges* provided relatively large-scale data sets, gaining massive attention from different research fields and greatly promoting the development of ML-based respiratory condition screening. The *ICBHI 2017 Challenge* released a database consisting of a total of 5.5 h of recordings containing 6898 respiratory cycles (i.e. from inspiratory to expiatory phase), of which 1864 contain crackles, 886 contain wheezes, and 506 contain both crackles and wheezes, in 920 annotated audio samples from 126 subjects. The recordings were collected using stethoscopes or microphones, and their duration ranged from 10 to 90 s. The chest locations from which the recordings were acquired are also provided. Participants were diagnosed with COPD (chronic obstructive pulmonary disease), LRTI (lower respiratory tract infection), or URTI (upper respiratory tract infection). Those cycles were annotated by respiratory experts. Therefore, this database can be used for either respiratory cycle-level sound classification or subject-level disease detection. In addition, *Pfizer Digital Medicine Challenge* created a respiratory disease database from other public audio databases. The open-source BMAT Annotation Tool was utilized to label whether an audio sample contains diseased sounds including coughing and sneezing. Finally, 2545 sick samples and 4048 non-sick samples were released for public use. Without specific respiratory abnormalities, *Pfizer* data can be used to train a cough or sneezing detector, which serves as a pre-prepossessing tool for the following respiratory condition screening task.

*Stethoscope* and *HF Lung V1* are additional lung sound databases. Lung sounds were acquired using multi-channel electronic stethoscopes placed on various vantage points of the chest wall. Subject ID with demographic information and recording location is provided. Respiratory cycles were manually annotated by specialists. *Stethoscope* consists of 336 recordings with varying lengths from 112 subjects, while *HF Lung V1* contains 9765 audio trunks with a length of 15 s from 279 subjects. These two recently released databases can be leveraged to validate the models developed via *ICBHI*, or ideally, those three databases can be jointly utilized to facilitate more promising ML algorithms for crackle and wheeze detection.

### COVID-19-related respiratory database

Since the outbreak of Coronavirus, researchers’ attention has been extended from crackle and wheeze detection to COVID-19 prediction, as Coronavirus can cause respiratory tract infections and inflammations, which may lead to audible changes to respiratory sounds. In recent years, several *COVID-19 audio databases* have been gathered.

Most COVID-19 audio databases collected cough sounds via microphones. Among those, *Virufy* is on the smallest scale with 121 recordings from 16 participants, but the COVID-19 status annotation is reliable as validated by clinical PCR (polymerase chain reaction). Another two larger COVID-19 databases with part of sample validated clinically are *Covid19-cough* and *COUGHVID*. The EPFL research team developed the *COUGHVID* database covering over 25,000 crowd-sourced cough recordings representing a wide range of participant ages, genders, geographic locations, and self-reported COVID-19 statuses, as well as subjects’ other respiratory conditions and symptoms (presenting or not). It is the largest cough database for the COVID-19 study. They also hired four respiratory experts to manually check the quality of audio recordings and the reported health status, but the proportion is small with only 4000 recordings confirmed. Compared to the above databases, *Tos COVID-19* is claimed as a fully clinically validated cough database. The acquisition of audio samples was done through WhatsApp from people who underwent a PCR or antigen swab test. In the released first version, 2821 individuals who were swabbed in the City of Buenos Aires between 11 August and 2 December 2020 were covered: 1409 tested positive for COVID-19 and 1412 tested negative. And a second data set containing 140,530 audio coughs was collected during the months of April to October 2021, with 18,271 audios from individuals who tested positive and 122,259 samples from negative individuals.

There are also two databases collecting cough as well as other sound types. In *Coswara*, for sound data, nine different categories, namely, breathing (two kinds; shallow and deep), cough (two kinds; shallow and heavy), sustained vowel phonation (three kinds; /ey/ as in made, /i/ as in beet, /u:/ as in cool), and 1–20 digit counting (two kinds; normal and fast-paced) were recorded. They also collected some metadata information, including age, gender, location (country and state), current health status (healthy, exposed, cured, or infected), and the presence of comorbidity (pre-existing medical conditions). The data collection is still ongoing, and as of the time we write this article, this database consists of 2747 samples with 681 represented as COVID-19 positive (can be asymptomatic, mild, or moderate). Similarly, *COVID-19 Sounds* database contains induced breathing, cough, and voice audio recordings. As samples in this database were collected through the app, subjects were assigned unique IDs. When participants registered the app, medical history, smoking status, and other general demographic information were collected. After that, participants could continually record their sounds and report their COVID-19 status. As a result, *COVID-19 Sounds* app is also able to collect longitudinal data that captures audio dynamics as well as COVID-19 status changes during a long period.^30^ In a nutshell, different sound types included in those two large-scale databases enable a comparison of the effectiveness of breathing, cough, and voice in detecting COVID-19; yet, the used COVID-19 statuses are self-reported without clinical validation.

### Database summary

Overall, more than 10 respiratory sound databases are publicly available for research. They are heterogeneous in terms of data acquiring protocol, associated respiratory conditions, and sound types. Some of them are crowd-sourced with self-reported health status from data contributors, while several of them are verified by experts. Those databases cover various sound types including lung sound, breathing, cough, and voice, as well as different respiratory conditions like asthma, COPD, and COVID-19. Nevertheless, data for asthma, COPD, and pertussis are still very limited: more databases covering those conditions are desired. Although high-quality audio samples with verified respiratory conditions are more reliable to use, considering the practical difficulty of collecting large-scale clinically validated data, jointly leveraging self-reported and physician-verified data can be efficient and effective. More collaborations among data scientists and respiratory experts can facilitate better data collection in the future.

## Methodology overview

**Figure 1.**
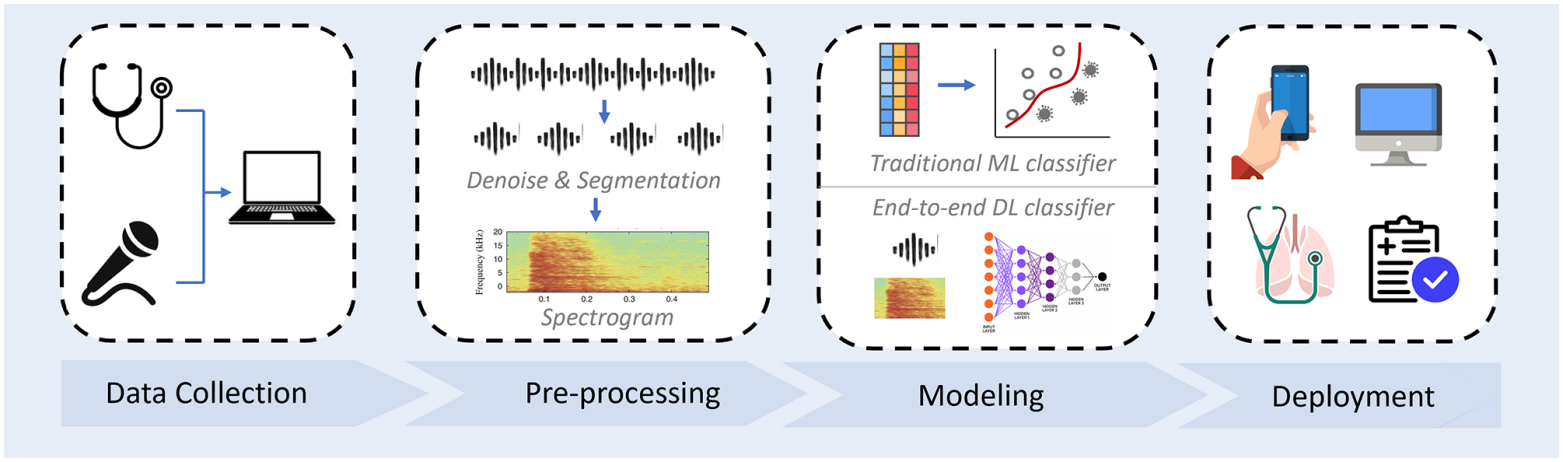
Automatic respiratory conditional screening system development pipeline. A typical system usually starts with audio data collection, followed by data pre-processing. Hand-crafted feature with traditional machine learning classification models or end-to-end deep learning models can be constructed. Before deployment to the public, the performance of the developed model needed to be validated on real-world clinical data. (A color version of this figure is available in the online journal.)

Audio-based respiratory condition screening can be formulated as a classification task, with the input of respiratory sounds and output of a categorical prediction for the trained respiratory conditions. Real-world collected audio signals can contain a variety of noises, and thus pre-processing before feeding them into ML models is needed. Audio signals are time series, characterized by not only temporal features but also spectral features in the transferred spectrograms. These features can be either explicitly utilized by traditional classifiers or implicitly explored by deep learning models. A typical automatic audio-based respiratory condition screening system development pipeline is illustrated in Figure 1. We have introduced existing databases in the previous section; in this section, we will introduce the commonly used pre-processing methods and compare the most representative models. It can be noted that features extracted from audio is known as physio-markers. Other diagnostic features like social-marker (e.g. subject demographics) and bio-marker (e.g. symptoms) are also informative,^31^ but we will mainly focus on the methods for physio-markers from sounds in this article.

### Pre-processing

Real-world collected audio samples are usually of low SNR (signal-to-noise ratio). For model development, proper de-noising is generally the first step before further processing. For lung sounds associated with crackle and wheeze, as suggested by the previous studies, re-sampling audio recordings to 4 KHz and deploying a fifth-order Butterworth band-pass filter having 100–200 Hz cut-off frequencies can effectively eliminate the environmental noise such as heartbeat, motion artifacts, and audio sounds.^32,33^ After that, respiratory cycles (inspiratory–expiatory periods) could be identified to further increase the SNR. Microphone-acquired audio data usually needs a sound-type check to avoid including improper sound modality, which can be performed manually or automatically.^34^ For instance, researchers developed a cough detector to select high-quality cough recordings for experiments.^26,28,29^ Some studies proposed to extract single cough clips from audio recordings as model inputs,^35^ as they think this further increases the SNR, while most researchers used the complete recordings because they hypothesize that silence frames between multiple coughs are also informative.^36,37^ Subsequently, temporal features can be extracted directly, and usually, audio segments will be transferred into spectrograms via short-time Fourier transforms. In addition, for microphone-recorded sounds, Mel scaling is commonly adopted for its unique capacity in modeling human listening characters.

Another important pre-processing step before model training is data augmentation for two purposes: first, most respiratory audio data sets are small and insufficient to train deep neural networks. Data augmentation can increase the data size for training. Widely used audio data augmentation methods include time stretch, pitch shift, perturbation, and noise injection on raw signals^38,39^ and masking or mix-up augmentation on spectrograms.^24,40,41^ Besides, the collected audio databases are class-imbalanced with a skewed distribution of the associated respiratory conditions. For example, COVID-19 databases have fewer COVID-19 positive samples than negative in Table 1. Such data imbalance makes it difficult to train a reliable classification model. To overcome this, data augmentation can be applied to additionally generate some samples for the minority classes to re-balance the data distribution. Up-sampling approaches like SMOTE are also widely used in addition to the above-mentioned methods.^42,43^

### Traditional ML models

Traditional ML-based auscultation models generally consist of two stages: (1) extracting acoustic features from audio signals and (2) training a classifier to predict the associated respiratory condition. We first introduce the developments of those two stages separately as below, and then we compare some reported performance on real-world repository data from the recent related literature.

Frequently explored respiratory acoustic features include temporal features such as onset, tempo, period, cross-zero-rate (CZR), beat-loudness, as well as spectral features like HNR, jitter, shimmer, Mel-frequency cepstral coefficients (MFCCs), spectral centroid, and roll-off frequency.^44,45^ There are many existing libraries that can be leveraged to automatically extract those features from raw signals, among which *Librosa* is a well-known Python-based programming tool.^46^ However, differences in audio signals associated with different respiratory conditions can be complex, subtle, and in-explicit, and thus, the above-mentioned features could be insufficient to distinguish various conditions. To this end, a number of statistical functionals have been proposed to extract massive high-order descriptors, such as the mean, delta, peak, and percentiles of those features across all frames of audio, showing favorable performance in many relevant tasks.^36^ openSMILE,^47^ MIRToolbox,^48^ and others are open-sourced tools for such feature set extraction, speeding up the processing procedure.

With such feature representation, a classifier – for example, DT (decision tree), RF (random forest), SVM (support vector machine), or MLP (multiple layer perceptron) – can be fitted for sound classification and respiratory disease prediction.^36,49^ DT is a classifier with tree-structured conditions to map features into several categories, and RF is the ensemble of DTs built with the bootstrapping of the training data to improve the resilience to errors.^17^ SVM is an algorithm that employs kernels to represent complex data in a low-dimensional and representative space, where it is desired to separate data belonging to every two clusters.^76^ For its flexible kernel selection and stable performance, SVM is the most widely marused method in the sound classification literature. MLP is an artificial neural network where features are fed into multiple layers with connection weights and activation functions. Weights are learnt via backpropagation, and thus, the model can well capture the relation between input features and the associated class.^76^

With the aforementioned features including MFCC, CZR, crest factor, energy level, and other 10 spectral features, and the SVM classifier, Pramono *et al.*^16^ achieved an accuracy of 100% in distinguishing 10 pertusses from 11 non-pertussis subjects. Similarly, the SVM classifier also showed an accuracy of around 99% in distinguishing COPD, pneumonia, and health subjects based on the International Conference on Biomedical and Health Informatics (ICBHI) 2017 database.^50^ In 2022, researchers further verified the promise that ML can be used to identify COPD subjects from healthy controls in a private but clinically validated voice data set, and according to their study, Compare2016 feature set developed by openSMILE toolkit presented better accuracy than other features.^77^ Based on the public ICBHI data base, Monaco *et al*.^76^ compared the performance of RF, MLP, and SVM by exploring 33 acoustic features with their statistics, although MLP yielded the highest accuracy of 85%, the performance difference from other models is marginal: their accuracy ranged from 81% to 85%. Overall, because of the light model with few parameters to fit, such hand-crafted feature-based traditional ML classifiers can usually achieve favorable performance and explainable classification, particularly when the audio database is not large.

### Deep learning models

With more audio data collected, deep learning, as part of a broader family of ML methods, has witnessed great progress in acoustic modeling.^19^ Because deep neural networks can significantly enhance the sound representation by capturing the complex relationship between the input audios and the output labels compared to the aforementioned hand-crafted features, deep learning usually yield better performance in various audio applications with a great promise shown in the respiratory condition screening domain.^49,51^

**Figure 2.**
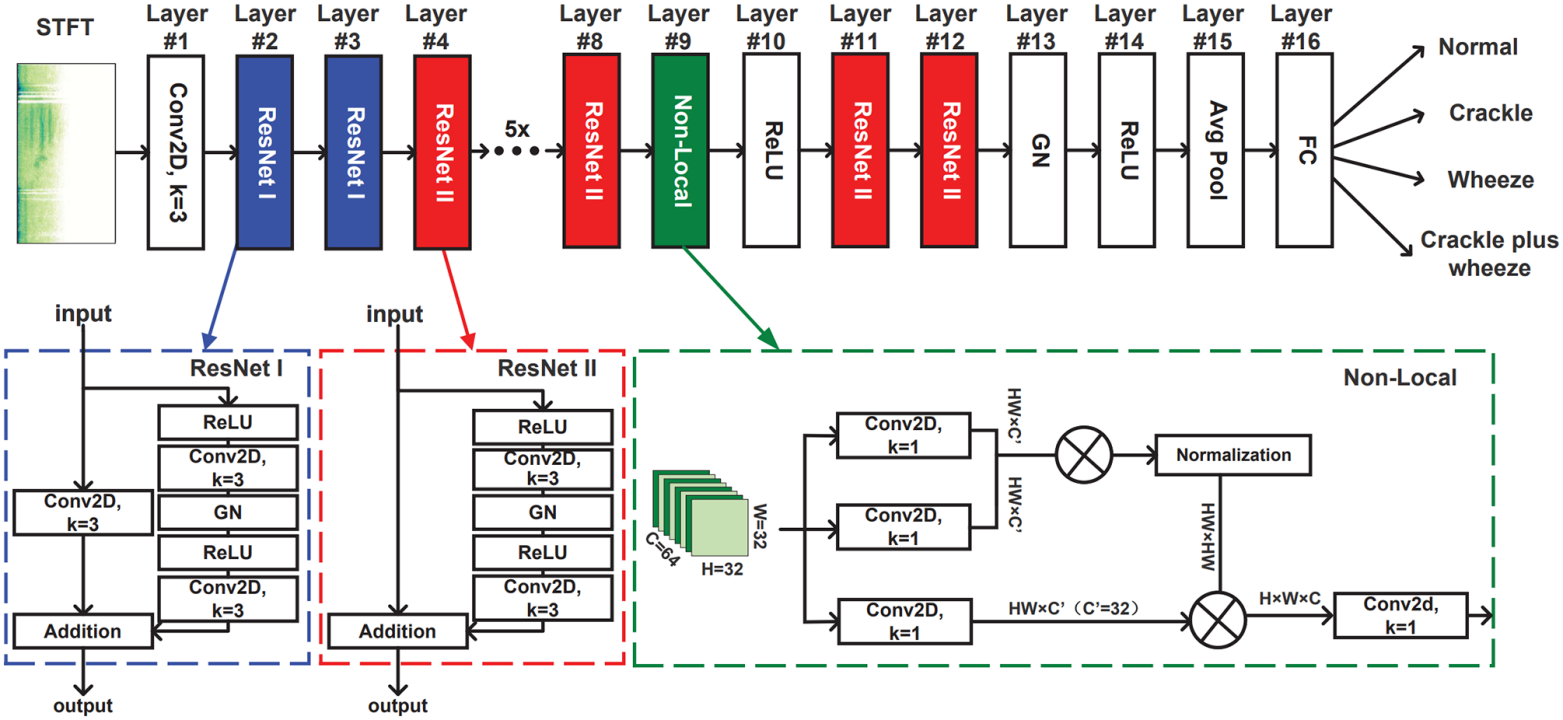
The proposed LungRN + NL neural network architecture for lung sounds classification used ICBHI 2017 database.^32^ This architecture consists of several ResNet and one non-local blocks. (A color version of this figure is available in the online journal.)

One typical acoustic deep learning model is the CNN based on spectrograms. Alike biological processes, the core mechanism of CNNs is that the connectivity pattern between neurons resembles the organization of the animal visual cortex. Individual neurons only respond to a small region the visual field, but multiple neurons can collectively cover the whole field. Inspired by the massive successes of CNNs in image classification tasks,^78^ exploring CNNs with spectrograms of audio signal as inputs for respiratory condition prediction has gained extensive attention as well as shown great potential. The promise of leveraging CNNs attributes to the power of CNN neurons which can capture complex spatial–temporal correlations in the spectrogram and to transfer the contextual information into distinguishable physio-markers for respiratory condition screening. The advance of CNNs has also been validated by experiments. Shi *et al.*^52^ devised CNN models to classify multiple lung sounds including wheeze, squawk, stridor, and crackle, reaching an accuracy over 95%.^53^ Variants of CNNs like VGGish^44,54^ and ResNet^32,55^ also have shown great performance in crackle detection, COPD prediction, and COVID-19 detection. An example of applying ResNet for crackle and wheeze classification is illustrated in Figure 2,^32^ where the ResNet layers can learn the characteristic of lung sounds through time and frequency domain, and the non-local layer between two ResNet layers can break the local time and frequency limit from the CNN. This model yielded an accuracy of 52.26% based on the official ICBHI 2017 challenge scoring standards, which is improved by 2.1%∼12.7% compared to the other models.

Another widely used deep learning technique for respiratory sound classification is RNN and its variants.^56–58^ Different from CNNs which equally treat frequency dimension and time dimension by two-dimensional (2D) conventional kernel neurons, RNNs utilize recurrent gate mechanisms to capture sequential pattern from the temporal context of audio signals. RNN can also overcome the restricted visual field of CNNs, leading to better cross-time and long-distance correlation modeling. Tiwari *et al.*^59^ developed a bi-directional RNN model via ICBHI 2017 database, yielding an accuracy of over 80% in detecting abnormal respiratory cycles. RNN can also be jointly applied with CNN model to better capture spatial–temporal features for respiratory sound classification.^60,61^

Similar to RNN, another sequential modeling architecture is Transformer, which has been explored recently for cough-based COVID-19 detection.^37,41,56,62,79^ Transformer treats audio spectrograms as token sequences with per spectrogram segment as one token. Benefiting from the attention mechanism, Transformer can learn a weighted combination of the features from different spectrogram segments: either close or far away, and thus, it is more capable of capturing the bio-markers that are embedded in the long audio signals. Experiments on the COVID-19 Sounds data base for the INTERSPEECH 2021 Computational Paralinguistics ChallengE (ComParE) have shown that the proposed Transformer-based model outperforms all other deep learning methods.^79^

Although most studies focus on sample-level condition prediction, there is some research jointly utilizing CNN and RNN on longitudinal audio data to model the respiratory abnormality progression.^30^ Dang *et al.* recently validated that the features captured by CNN from respiratory sound spectrograms showed a close correlation with the subject’s COVID-19 recovery process, and leveraging RNN based on those features can predict the COVID-19 status timely and accurately. Such investigation can further extend the value of digital respiratory health for early diagnosis and treatment.

### Method summary

Various model architectures have been explored on respiratory audio data, which show promising performance for automatic respiratory condition screening. However, the transparency of implementation details is lacking, and some models are developed based on private databases with no codes published. For real-world deployment, further validation of the model performance on clinically verified data is necessary to avoid over-fitting on experimental data. Data scientists are expert in modeling while respiratory physicians have their domain knowledge in feature designing and performance valuation, and thus, more in-depth cooperation beyond data collection is desired and crucial for high-performance respiratory condition screening systems.

## Open issues

In spite of the massive efforts that have greatly advanced the development of automatic respiratory condition screening, there are still a plethora of challenges unsolved, and those open issues are worth exploring.

### Lack of data

Reliable and large-scale databases are a bottleneck for ML-based applications. As we summarize in the data overview section, many respiratory conditions are not covered by existing publicly available audio databases. Even for the widely studied COVID-19 disease, some databases are crowd-sourced without clinical verification. Considering the sensitivity of health screening, models developed by such data need careful validation before deployment. Combining different databases to extend the data for model training might be a potential solution for the limited data; however, given the high heterogeneity of those public databases, it is very challenging. In addition to putting efforts to collect more data, for model developers, small-data learning techniques including semi-supervised learning,^63^ self-supervised learning,^64^ and transfer learning^65^ can be explored. For example, warming up the model training by leveraging non-respiratory audio data or non-labeled respiratory audio data and then transferring the model to the target auscultation task can be helpful.^41,69,80^ When new respiratory audio data continuously become accessible, incremental learning,^66^ meta learning,^67^ and active learning approaches^68^ can be applied to subsequently improve existing models.

### Better interpretability

ML particularly deep learning models are known as black boxes, lacking proper interpretation of how the prediction is made. Yet, for clinical use, a well understanding of what kind of physio-markers are leveraged is of great importance to avoid decision bias for diagnosis. For example, spoken language should not be used as a feature for respiratory condition screening, but this information is easy to be captured by the model and sometimes could be explicitly misused in experiments with biased data distribution.^69^ Post hoc interpretation with a holistic evaluation of the developed models is requested but under-looked in the current literature. For ML methods, acoustic feature importance should be derived to seek the meaning and explanation with the associated respiratory condition in the real world.^81^ On the other side, attention mechanism could be a plausible option to be incorporated in deep neural networks so that the significance of different spectrogram segments can be traced.^41,79^

### Risk management

Another important issue for ML, particularly deep learning, based health applications is risk management. Although they show promising results in the laboratory-collected data, commonly used deep learning models can be poorly calibrated,^70^ yielding overconfident predictive probabilities which cannot reflect the true diagnostic confidence of diagnosis in the wild. Deep learning also behaves unpredictably on unfamiliar data, for example, unseen sound-type, new respiratory conditions, noising audio signal inputs, which has profound effects in the clinical context.^71^ Those misdiagnosis risks should be well managed, and when ML cannot handle them, physicians can be involved in time. A potential solution is to quantify the prediction uncertainty of the acoustic models, which can raise a warning for unfamiliar audio inputs and unconfident respiratory condition predictions.^72–74^

### Privacy preservation

Health data are always sensitive. When collecting health data for diagnostic system development, user privacy has been a persistent concern. Privacy-preserving deep modeling attempts to bridge the gap between personal data protection and data usage for clinical routine and research, thus being a promising solution. The privacy-preserving mechanisms can be applied to the whole deep modeling chain, from data acquisition, through model training, to model inference.^75^ Federated learning, which can train models collectively with the data remained on the contributors’ side, is widely adapted in various health applications.^82,83^ Although little work has been done in this respective for automatic auscultation, lessons can be learnt from related tasks including acoustic event classification, audio recognition, and so on.^84–86^

## Conclusions

In this concise review, we present the advance and promise of exploring ML for respiratory condition screening. AI-powered auscultation via respiratory sounds collected by electronic stethoscopes and microphones has great flexibility and scalability: the screening can be done remotely, and the results can be delivered to users by smartphones, expediting medical diagnosis outside the hospital. To facilitate the development of automatic respiratory condition screening systems, we summarize more than 10 publicly available audio databases covering various respiratory conditions and discuss several representative features as well as architecture designing approaches for respiratory sound modeling. Those latest techniques have shown favorable performance in some contexts; however, there are still many open issues that are needed to be solved before deploying the developed models to the public. Specially, we point out that small-data learning, interpretable features, uncertainty-aware models, and privacy-preservation prediction are worth exploring in future work to handle the unsolved challenges.

## References

[1] Fincham W Tehrani F . A mathematical model of the human respiratory system. J Biomed Eng 1983;5:125–33 10.1016/0141-5425(83)90030-4 6406766

[2] Childers DG Hicks D Moore G Eskenazi L Lalwani A . Electroglottography and vocal fold physiology. J Speech Lang Hear Res 1990;33:245–54 10.1044/jshr.3302.245 2359265

[3] Martin SA Pence BD Woods JA . Exercise and respiratory tract viral infections. Exerc Sport Sci Rev 2009;37:157 19955864 10.1097/JES.0b013e3181b7b57b PMC2803113

[4] Cappelletty D . Microbiology of bacterial respiratory infections. Pediatr Infect Dis J 1998;17:S55–61 10.1097/00006454-199808001-00002 9727651

[5] Burki TK . The economic cost of respiratory disease in the UK. Lancet Respir Med 2017;5:381 28341204 10.1016/S2213-2600(17)30108-X

[6] Wood CS Thomas MR Budd J Mashamba-Thompson TP Herbst K Pillay D Peeling RW Johnson AM McKendry RA Stevens MM. Taking connected mobile-health diagnostics of infectious diseases to the field. Nature 2019;566:467–74 10.1038/s41586-019-0956-2 PMC6776470 30814711

[7] Collins FS Varmus H. A new initiative on precision medicine. N Engl J Med 2015;372:793–5 10.1056/NEJMp1500523 PMC5101938 25635347

[8] Shaban-Nejad A Michalowski M Buckeridge DL. Health intelligence: how artificial intelligence transforms population and personalized health. NPJ Digit Med 2018;1:1–2 31304332 10.1038/s41746-018-0058-9 PMC6550150

[9] Ramkumar PN Haeberle HS Bloomfield MR Schaffer JL Kamath AF Patterson BM Patterson BM Krebs VE. Artificial intelligence and arthroplasty at a single institution: real-world applications of machine learning to big data, value-based care, mobile health, and remote patient monitoring. J Arthroplasty 2019;34:2204–9 10.1016/j.arth.2019.06.018 31280916

[10] Amiriparian S Schuller B . AI hears your health: computer audition for health monitoring. In: Proceedings of the conference on health and wellbeing, 2021, pp.227–33, https://link.springer.com/chapter/10.1007/978-3-030-94209-0_20

[11] Schuller BW Schuller DM Qian K Liu J Zheng H Li X. COVID-19 and computer audition: an overview on what speech & sound analysis could contribute in the SARSCoV-2 corona crisis. Front Digit Heal 2021; 3:14 10.3389/fdgth.2021.564906 PMC8521916 34713079

[12] Keating T. ResApp technology to diagnose and manage respiratory disease. Australas Biotechnol 2015;25:16

[13] Hadjitodorov S Mitev P. A computer system for acoustic analysis of pathological voices and laryngeal diseases screening. Med Eng Phys 2002;24:419–29 10.1016/s1350-4533(02)00031-0 12135650

[14] Mukherjee H Sreerama P Dhar A Obaidullah SM Roy K Mahmud M Santosh KC. Automatic lung health screening using respiratory sounds. J Med Sys 2021;45:1–9 10.1007/s10916-020-01681-9 PMC7797201 33426615

[bibr15-15353702221115428] Srivastava A Jain S Miranda R Patil S Pandya S Kotecha K. Deep learning based respiratory sound analysis for detection of chronic obstructive pulmonary disease. PeerJ Comput Sci 2021;7:1–22 10.7717/peerj-cs.369 PMC7959628 33817019

[bibr16-15353702221115428] Pramono RXA Imtiaz SA Rodriguez-Villegas E . A cough-based algorithm for automatic diagnosis of pertussis. PLoS ONE 2016;11:e0162128 10.1371/journal.pone.0162128 PMC5008773 27583523

[17] Hao T Xing G Zhou G. isleep: unobtrusive sleep quality monitoring using smartphones. In: Proceedings of the ACM conference on ENSS, 2013, pp.1–14, https://www.cs.wm.edu/~gzhou/files/iSleep_SenSys13.pdf

[18] Schuller B . Chain of audio processing. Intell Audio Analy 2013;1:17–22

[19] Yu D Li J. Recent progresses in deep learning based acoustic models. IEEE/CAA J Autom Sin 2017;4:396–409

[20] Rocha BM Filos D Mendes L Serbes G Ulukaya S Kahya YP Jakovljevic N Turukalo TL Vogiatzis IM Perantoni E Kaimakamis E Natsiavas P Oliveira A Jácome C Marques A Maglaveras N Paiva RP Chouvarda I de Carvalho P . An open access database for the evaluation of respiratory sound classification algorithms. Physiol Meas 2019;40:035001 30708353 10.1088/1361-6579/ab03ea

[21] Piczak KJ . ESC: dataset for environmental sound classification. In: Proceedings of the ACM conference on MM, 2015, pp.1015–18, https://dataverse.harvard.edu/dataset.xhtml?persistentId=doi:10.7910/DVN/YDEPUT

[22] Fraiwan M Fraiwan L Khassawneh B Ibnian A. A dataset of lung sounds recorded from the chest wall using an electronic stethoscope. Data Brief 2021;35:106913 33732827 10.1016/j.dib.2021.106913 PMC7937981

[23] Hsu FS Huang SR Huang CW Huang CJ Cheng YR Chen CC Hsiao J Chen C-W Chen L-C Lai Y-C Hsu B-F Lin N-J Tsai W-L Lai F. Benchmarking of eight recurrent neural network variants for breath phase and adventitious sound detection on a self-developed open-access lung sound database: HF Lung V1. PLoS ONE 2021;16:e0254134 10.1371/journal.pone.0254134 PMC8248710 34197556

[24] Belkacem AN Ouhbi S Lakas A Benkhelifa E Chen C. End-to-end AI-based point-of-care diagnosis system for classifying respiratory illnesses and early detection of COVID-19: a theoretical framework. Front Med 2021;8:372 10.3389/fmed.2021.585578 PMC8044874 33869239

[25] Ponomarchuk A Burenko I Malkin E Nazarov I Kokh V Avetisian M Zhukov L. Project Achoo: a practical model and application for COVID-19 detection from recordings of breath, voice, and cough. IEEE J Sel Top in Signal Process 2022;16:175–187 35582703 10.1109/JSTSP.2022.3142514 PMC9088778

[26] Orlandic L Teijeiro T Atienza D. The COUGHVID crowdsourcing dataset, a corpus for the study of large-scale cough analysis algorithms. Sci Data 2021;8:1–10 34162883 10.1038/s41597-021-00937-4 PMC8222356

[27] Johnsi R Kumar GB Sariki TP. A concise survey on datasets, tools and methods for biomedical text mining. Int J Appl Eng Res 2022;17:200–17

[28] Sharma N Krishnan P Kumar R Ramoji S Chetupalli S Nirmala R Ghosh PK Ganapathy S . Coswara: a database of breathing, cough, and voice sounds for COVID-19 diagnosis. In: Proceedings of the conference on INTERSPEECH, 2020, pp.4811–5, https://arxiv.org/abs/2005.10548

[29] Xia T Spathis D Ch J Grammenos A Han J Hasthanasombat A Bondareva E Dang T Floto A Cicuta P Mascolo C . COVID-19 sounds: a large-scale audio dataset for digital respiratory screening. In: Proceedings of the NeurIPS, 2021, pp.1–13, https://datasets-benchmarks-proceedings.neurips.cc/paper/2021/file/e2c0be24560d78c5e599c2a9c9d0bbd2-Paper-round2.pdf

[30] Dang T Han J Xia T Spathis D Bondareva E Siegele-Brown C Chauhan J Grammenos A Hasthanasombat A Floto RA Cicuta P . Exploring Longitudinal Cough, Breath, and Voice Data for COVID-19 Progression Prediction via Sequential Deep Learning: model Development and Validation. J Med Inter Res 2022;24:1–35 10.2196/37004 PMC9217153 35653606

[31] Palaniyappan L . More than a biomarker: could language be a biosocial marker of psychosis? NPJ Schizophr 2021;7:1–5 34465778 10.1038/s41537-021-00172-1 PMC8408150

[32] Ma Y Xu X Li Y. LungRN+ NL: an improved adventitious lung sound classification using non-local block ResNet neural network with Mixup data augmentation. In: Proceedings of the conference INTERSPEECH, 2020, pp.2902–6, https://researchr.org/publication/MaXL20-1

[33] Minami K Lu H Kim H Mabu S Hirano Y Kido S . Automatic classification of largescale respiratory sound dataset based on convolutional neural network. In: Proceedings of the conference on control, automation and systems (ICCAS), Jeju, South Korea, 15–18 October 2019, pp.804–7. New York: IEEE.

[34] Pramono RXA Imtiaz SA Rodriguez-Villegas E . Automatic cough detection in acoustic signal using spectral features. In: Proceedings of the 2019 41st annual international conference of the IEEE engineering in medicine and biology society (EMBC), Berlin, 23–27 July 2019, pp.7153–6. New York: IEEE. 10.1109/EMBC.2019.8857792 31947484

[35] Swarnkar V Abeyratne UR Amrulloh Y Hukins C Triasih R Setyati A. Neural network based algorithm for automatic identification of cough sounds. Proc IEEE Conf Eng Med Biol Sci 2013;2013:1764–7 10.1109/EMBC.2013.6609862 24110049

[36] Schuller BW Batliner A Bergler C Mascolo C Han J Lefter I Kaya H Amiriparian S Baird A Stappen L Ottl S Gerczuk M Tzirakis P Brown C Chauhan J Grammenos A Hasthanasombat A Spathis D Xia T Cicuta P Rothkrantz LJM Zwerts J Treep J Kaandorp C. The INTERSPEECH 2021 computational paralinguistics challenge: COVID-19 cough, COVID-19 speech, escalation & primates. In: Proceedings of the conference on INTERSPEECH, 2021, pp.431–5, https://arxiv.org/abs/2102.13468

[37] Qian K Schuller BW Yamamoto Y . Recent advances in computer audition for diagnosing COVID-19: an overview. In: Proceedings of the 2021 IEEE 3rd global conference on life sciences and technologies (LifeTech), Nara, Japan, 9–11 March 2021, pp.181–2. New York: IEEE.

[38] Zhou Q Shan J Ding W Wang C Yuan S Sun F Li H Fang B. Cough recognition based on Mel-spectrogram and convolutional neural network. Front Robot AI 2021;8:580080 34026854 10.3389/frobt.2021.580080 PMC8138471

[39] Yella N Rajan B. Data augmentation using GAN for sound based COVID 19 diagnosis. Proc IEEE Conf IDAACS 2021;2:606–9

[40] Gairola S Tom F Kwatra N Jain M . RespireNet: a deep neural network for accurately detecting abnormal lung sounds in limited data setting. In: Proceedings of the IEEE conference on EMBS, 2021, pp.527–30, https://arxiv.org/abs/2011.00196 10.1109/EMBC46164.2021.9630091 34891348

[41] Xue H Salim FD . Exploring self-supervised representation ensembles for Covid-19 cough classification. In: Proceedings of the ACM conference on KDD, 2021, pp.1944–52, https://arxiv.org/abs/2105.07566

[42] Han J Brown C Chauhan J Grammenos A Hasthanasombat A Spathis D Xia T Cicuta P Mascolo C. Exploring automatic COVID-19 diagnosis via voice and symptoms from crowdsourced data. In: Proceedings of the IEEE conference on ICASSP, 2021, pp.8328–32, https://arxiv.org/abs/2102.05225

[43] Pahar M Klopper M Warren R Niesler T. COVID-19 cough classification using machine learning and global smartphone recordings. Comput Biol and Med 2021;135:104572 34182331 10.1016/j.compbiomed.2021.104572 PMC8213969

[44] Brown C Chauhan J Grammenos A Han J Hasthanasombat A Spathis D Xia T Cicuta P Mascolo C . Exploring automatic diagnosis of COVID-19 from crowdsourced respiratory sound data. In: Proceedings of the ACM conference on KDD, 2020, pp.3474–84, https://arxiv.org/abs/2006.05919

[45] Chambres G Hanna P Desainte-Catherine M . Automatic detection of patient with respiratory diseases using lung sound analysis. In: Proceedings of the 2018 international conference on content-based multimedia indexing (CBMI), La Rochelle, 4–6 September 2018, pp.1–6. New York: IEEE.

[46] McFee B Raffel C Liang D Ellis DP McVicar M Battenberg E Nieto O. librosa: audio and music signal analysis in python. Python Sci Conf 2015;8:18–25

[47] Eyben F Wöllmer M Schuller B . Opensmile: the munich versatile and fast open-source audio feature extractor. In: Proceedings of the 18th ACM international conference on multimedia, Firenze, 25–29 October 2010, pp.1459–62. New York: ACM.

[48] Lartillot O Toiviainen P Eerola T . A Matlab toolbox for music information retrieval. In: Data analytics and machine learning applications, 2008, pp.261–68, https://citeseerx.ist.psu.edu/viewdoc/download?doi=10.1.1.706.2450&rep=rep1&type=pdf#:~:text=MIRToolbox%20is%20a%20Matlab%20toolbox,be%20applied%20to%20statistical%20analyses.

[49] Ullah A Khan MS Khan MU Mujahid F . Automatic classification of lung sounds using machine learning algorithms. In: Proceedings of the 2021 international conference on Frontiers of information technology (FIT), Islamabad, Pakistan, 13–14 December 2022, pp.131–36. New York: IEEE.

[50] Naqvi SZH Choudhry MA. An automated system for classification of chronic obstructive pulmonary disease and pneumonia patients using lung sound analysis. Sensors 2020;20:6512 33202613 10.3390/s20226512 PMC7697014

[51] Coppock H Gaskell A Tzirakis P Baird A Jones L Schuller B. End-to-end convolutional neural network enables COVID-19 detection from breath and cough audio: a pilot study. BMJ Innova 2021;7:356–62 10.1136/bmjinnov-2021-000668 34192022

[52] Shi L Du K Zhang C Ma H Yan W. Lung sound recognition algorithm based on VGGish-BiGRU. IEEE Access 2019;7:139438–49

[53] Bardou D Zhang K Ahmad SM. Lung sounds classification using convolutional neural networks. Artif Intell Med 2018;88:58–69 29724435 10.1016/j.artmed.2018.04.008

[54] Demir F Sengur A Bajaj V. Convolutional neural networks based efficient approach for classification of lung diseases. Heal Inf Sci and Sys 2020;8:1–8 10.1007/s13755-019-0091-3 PMC6928168 31915523

[55] Laguarta J Hueto F Subirana B. COVID-19 artificial intelligence diagnosis using only cough recordings. IEEE J Eng Med Biol 2020;1:275–81 10.1109/OJEMB.2020.3026928 PMC8545024 34812418

[56] Deshpande G Batliner A Schuller BW. AI-based human audio processing for COVID19: a comprehensive overview. Pattern Recogn 2022;122:108289 10.1016/j.patcog.2021.108289 PMC8404390 34483372

[bibr57-15353702221115428] Rocha BM Pessoa D Marques A Carvalho P Paiva RP. Automatic classification of adventitious respiratory sounds: a (un)solved problem? Sensors 2020;21:57 33374363 10.3390/s21010057 PMC7795327

[58] Tabatabaei SAH Fischer P Schneider H Koehler U Gross V Sohrabi K . Methods for adventitious respiratory sound analyzing applications based on smartphones: a survey. IEEE Rev Bio Eng 2020;14:98–115 10.1109/RBME.2020.3002970 32746364

[59] Tiwari U Bhosale S Chakraborty R Kopparapu SK . Deep lung auscultation using acoustic biomarkers for abnormal respiratory sound event detection. In: ICASSP 2021: 2021 IEEE international conference on acoustics, speech and signal processing (ICASSP), Toronto, ON, Canada, 6–11 June 2021, pp.1305–9. New York: IEEE.

[60] Rashid HA Mazumder AN Niyogi UPK Mohsenin T . CoughNet: a flexible low power CNN-LSTM processor for cough sound detection. In: Proceedings of the 2021 IEEE 3rd international conference on artificial intelligence circuits and systems (AICAS), Washington, DC, 6–8 June 2021, pp.1–4. New York: IEEE.

[61] Perna D Tagarelli A. Deep auscultation: predicting respiratory anomalies and diseases via recurrent neural networks. Proc Symp CMS 2019:50–5, https://arxiv.org/abs/1907.05708

[62] Chang Y Ren Z Schuller BW. Transformer-based CNNs: mining temporal context information for multi-sound COVID-19 diagnosis. Proc IEEE Conf EMBS 2021;2021:2335–8 10.1109/EMBC46164.2021.9629552 34891751

[63] Van Engelen JE Hoos HH. A survey on semi-supervised learning. Mach Learn 2020;109:373–440

[64] Jaiswal A Babu AR Zadeh MZ Banerjee D Makedon F. A survey on contrastive self-supervised learning. Technol 2020;9:2

[65] Pan SJ Yang Q. A survey on transfer learning. IEEE Trans Knowl Data Eng 2009;22:1345–59

[66] Liu X Wu C Menta M Herranz L Raducanu B Bagdanov AD Jui S de Weijer JV . Generative feature replay for class-incremental learning. In: Proceedings of the IEEE/CVF conference on CVPR, 2020, pp.226–227, https://arxiv.org/abs/2004.09199

[67] Hospedales TM Antoniou A Micaelli P Storkey AJ. Meta-learning in neural networks: a survey. IEEE Trans Pattern Anal Mach Intell 2021; 1:3079209 10.1109/TPAMI.2021.3079209 33974543

[68] Aggarwal CC Kong X Gu Q Han J Philip SY. Active learning: a survey. In: Data classification, 2014, pp.599–634, http://charuaggarwal.net/active-survey.pdf

[69] Han J Xia T Spathis D Bondareva E Brown C Chauhan J Dang T Grammenos A Hasthanasombat A Floto A Cicuta P Mascolo C. Sounds of COVID-19: exploring realistic performance of audio-based digital testing. NPJ Digit Med 2022;5:1–9 35091662 10.1038/s41746-021-00553-x PMC8799654

[70] Guo C Pleiss G Sun Y Weinberger KQ . On calibration of modern neural networks. In: Proceedings of the conference on ML, 2017, pp.1321–30, https://arxiv.org/abs/1706.04599

[71] Ovadia Y Fertig E Ren J Nado Z Sculley D Nowozin S Dillon JV Lakshminarayanan B Snoek J. Can you trust your model’s uncertainty? Evaluating predictive uncertainty under dataset shift. In: Proceedings of the conference on NeurIPS, 2019, pp.3991–4002, https://arxiv.org/abs/1906.02530

[72] Xia T Han J Qendro L Dang T Mascolo C. Uncertainty-aware covid-19 detection from imbalanced sound data. In: Proceedings of the conference on INTERSPEECH, 2021, pp.216–20, https://arxiv.org/abs/2104.02005

[bibr73-15353702221115428] Xia T Han J Mascolo C . Benchmarking uncertainty quantification on biosignal classification tasks under dataset shift. In: Proceedings of the workshop heal intelligence, 2022, pp.1–10, https://arxiv.org/abs/2112.09196

[74] Park C Awadalla A Kohno T Patel S . Reliable and trustworthy machine learning for health using dataset shift detection. In: Proceedings of the conference on NeurIPS, 2021, pp.1–13, https://ubicomplab.cs.washington.edu/pdfs/mhealth_ood.pdf

[75] Kaissis GA Makowski MR Rückert D Braren RF. Secure, privacy-preserving and federated machine learning in medical imaging. Nat Mach Intell 2020;2:305–11

[76] Monaco A Amoroso N Bellantuono L Pantaleo E Tangaro S Bellotti R . Multi-time-scale features for accurate respiratory sound classification. Appl Sci.2020;10:8606

[77] Nallanthighal VS Härmä A Strik H . Detection of COPD exacerbation from speech: comparison of acoustic features and deep learning based speech breathing models. In: Proceedings of the IEEE international conference on ICASSP. Singapore, 23–27 May 2022, pp.97–101. New York: IEEE.

[78] Dhruv P Naskar S. Image classification using convolutional neural network (CNN) and recurrent neural network (RNN): a review. Mach Learn Inf Process 2020;1:367–81

[79] Yan T Meng H Liu S Parada-Cabaleiro E Ren Z Schuller BW . Convoluational transformer with adaptive position embedding for Covid-19 detection from cough sounds. In: Proceedings of the ICASSP 2022: 2022 IEEE international conference on acoustics, speech and signal processing (ICASSP), Singapore, 23–27 May 2022, pp.92–96. New York: IEEE.

[80] Chen XY Zhu QS Zhang J Dai LR. Supervised and self-supervised pretraining based COVID-19 detection using acoustic breathing/cough/speech signals. In: Proceedings of the ICASSP 2022: 2022 IEEE international conference on acoustics, speech and signal processing (ICASSP), Singapore, 23–27 May 2022, pp.561–65. New York: IEEE.

[81] Carvalho DV Pereira EM Cardoso JS. Machine learning interpretability: a survey on methods and metrics. Electronics 2019;8:832

[82] Rieke N Hancox J Li W Milletari F Roth HR Albarqouni S Bakas S Galtier MN Landman BA Maier-Hein K Ourselin S. The future of digital health with federated learning. NPJ Digit Med 2020;14:1–7 10.1038/s41746-020-00323-1 PMC7490367 33015372

[83] Xu J Glicksberg BS Su C Walker P Bian J Wang F . Federated learning for healthcare informatics. J Healthc Inform Res.2021;5:1–9 33204939 10.1007/s41666-020-00082-4 PMC7659898

[84] Gao Y Parcollet T Zaiem S Fernandez-Marques J de Gusmao PP Beutel DJ Lane ND. End-to-end speech recognition from federated acoustic models. In: Proceedings of the ICASSP 2022: 2022 IEEE international conference on acoustics, speech and signal processing (ICASSP), Singapore, 23–27 May 2022, pp.27–31. New York: IEEE.

[bibr85-15353702221115428] Feng M Kao CC Tang Q Sun M Rozgic V Matsoukas S Wang C . Federated self-supervised learning for acoustic event classification. In: Proceedings of the ICASSP 2022: 2022 IEEE international conference on acoustics, speech and signal processing (ICASSP), Singapore, 23–27 May 2022, pp.481–5. New York: IEEE.

[86] Tsouvalas V Saeed A Ozcelebi T . Federated self-training for data-efficient audio recognition. In: Proceedings of the ICASSP 2022: 2022 IEEE international conference on acoustics, speech and signal processing (ICASSP), Singapore, 23–27 May 2022, pp.476–80. New York: IEEE.

